# Emergency department diagnosis of upper extremity deep venous thrombosis using bedside ultrasonography

**DOI:** 10.1186/2036-7902-4-4

**Published:** 2012-04-16

**Authors:** Tony Rosen, Betty Chang, Martha Kaufman, Mary Soderman, David C Riley

**Affiliations:** 1 Emergency Medicine Department, Columbia University Medical Center, New York, NY, 10032, USA

**Keywords:** ultrasound, upper extremity deep venous thrombosis, color doppler.

## Abstract

A 27-year-old man presents to the emergency department with a 1-day history of severe right upper extremity pain and swelling. The patient's status is post open reduction internal fixation for a left tibial plateau fracture, which was complicated by methicillin-sensitive *Staphylococcus aureus *osteomyelitis. A peripherally inserted central catheter (PICC) line was subsequently placed for intravenous antibiotic therapy. Emergency department bedside ultrasound examination of both the right axillary vein and subclavian vein near the PICC line tip revealed deep venous thrombosis of both veins. Bedside upper extremity vascular ultrasonography can assist in the rapid diagnosis of upper extremity deep venous thrombosis in the emergency department.

## Background

Until recently, there has been much less clinical and research focus on identification and management of upper extremity deep venous thromboses (UEDVTs) than deep venous thromboses (DVTs) of the lower extremity. Historically, UEDVTs were believed to be quite rare, representing less than 2% of DVTs [1], and to be clinically insignificant if they occurred [1,2]. More recent research is challenging both of these beliefs. Recent studies suggest that upper extremity thrombi represent 10% to 18% of DVT cases [3,4] and are increasing as use of indwelling catheters and pacemakers increases. The long-held notion that UEDVTs generally have a benign, self-limited course and very seldom cause pulmonary embolism [1,2] has also been recently challenged. While the actual accurate prevalence of pulmonary embolism due to UEDVT is not known, research has found that pulmonary embolism may occur in 7% to 17% of upper extremity DVTs [5-7].

## Case presentation

**Figure 1:**
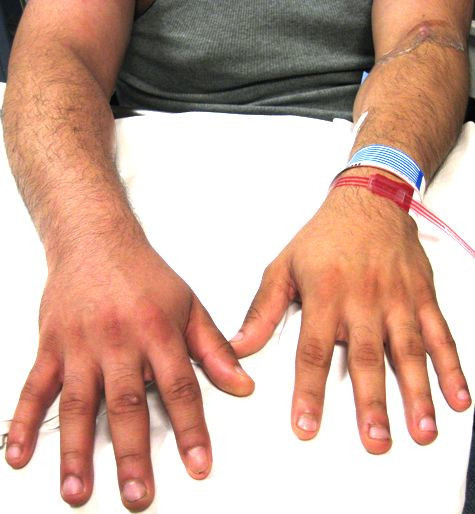
**Right upper extremity edema due to axillary and subclavian thrombosis**.

A 27-year-old man presents to the emergency department with a 1-day history of severe right upper extremity pain and swelling. The patient's status is post open reduction internal fixation for a left tibial plateau fracture, which was complicated by methicillin-sensitive *Staphylococcus aureus *osteomyelitis. A peripherally inserted central catheter (PICC) line was subsequently placed for intravenous antibiotic therapy. He denied any other past medical history. Aside from the pain and swelling to his right arm, the patient reports no associated shortness of breath, chest pain, or fever. His emergency department (ED) vital signs were as follows: temperature 97.1°F, blood pressure 135/74 mmHg, heart rate 82 bpm, respiratory rate 14 bpm, and oxygen saturation 100% on room air. Physical examination result was normal except for the right upper extremity that showed surrounding edema and mild erythema by the PICC line site (Figure 1). The arm had tenderness to palpation, but no significant increase in warmth was noted.

**Figure 2:**
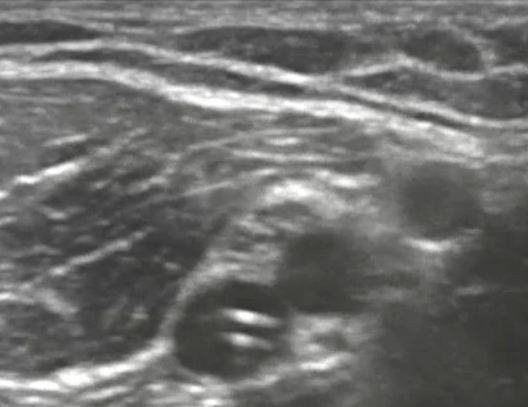
**Short-axis view of emergency department ultrasonography evaluation of PICC line in the brachial vein**.

**Figure 3:**
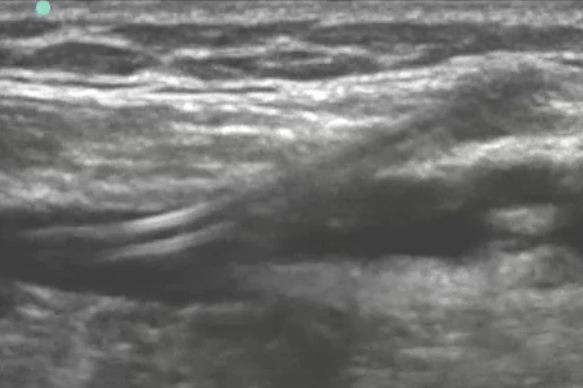
**Long-axis view of emergency department ultrasonography evaluation of PICC line in the brachial vein**.

**Figure 4:**
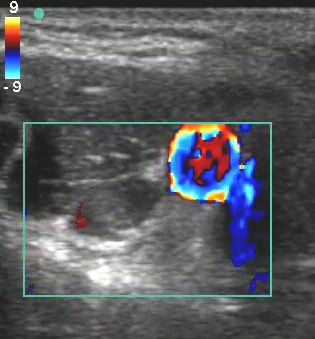
**Short-axis view of emergency department color Doppler ultrasonography of an axillary vein thrombosis**.

**Figure 5:**
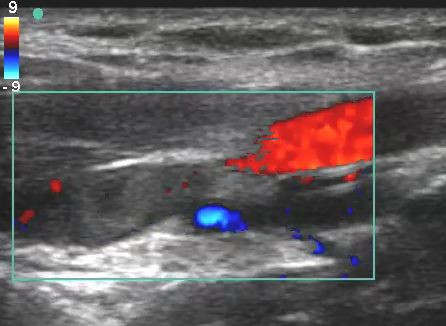
**Long-axis view of emergency department color Doppler ultrasonography of an axillary and subclavian vein thrombosis**.

On initial assessment, the triage nurse suspecting a possible upper extremity deep vein thrombosis notified the ED physician to facilitate a rapid bedside ultrasound of the right upper extremity. This ultrasound was performed (Additional files 1, 2, 3, and 4, available in the online version of this paper). Both short-axis and long-axis views of the brachial vein are shown (Figures 2 and 3 and Additional files 1, 2). The short-axis view of the right axillary vein revealed a non-compressible deep venous thrombosis (Figure 4 and Additional file 3). Long-axis ultrasonographic evaluation of the axillary and subclavian veins near the PICC line tip revealed deep venous thrombosis of both the axillary and subclavian veins (Figure 5 and Additional file 4).

Comprehensive radiology ultrasonography of the right upper extremity showed no flow and/or compressibility in the right subclavian vein or the right axillary vein adjacent to the PICC line, consistent with complete thrombosis. The right internal jugular vein and right brachial vein were patent and compressible, and the right innominate vein demonstrated patent flow. The patient was admitted to the hospital, and he was treated with oral antibiotics and subcutaneous enoxaparin injections.

## Discussion

Upper extremity DVT may be classified into primary and secondary subtypes based on the pathogenesis [3,8]. Primary UEDVT, Paget-Schrötter syndrome, was first described independently by Sir James Paget and Leopold von Schrötter in the nineteenth century [9-11]. Often called 'effort' thrombosis, it is a UEDVT occurring spontaneously after strenuous occupational or recreational activity, such as scraping wallpaper, throwing a baseball, or weightlifting [8,11,12]. This heavy exertion is thought to cause microtrauma to the vessel intima because of repeated mechanical compression from the clavicle, first rib, and enlarged shoulder muscles [3,11]. It occurs most commonly in young, healthy men and is more commonly found in the right arm, likely because this is usually dominant and involved in more strenuous activity [10]. This syndrome is uncommon, but it should be considered by emergency physicians when presented with arm pain and swelling in an otherwise healthy adult and is particularly relevant for physicians focusing on sports or occupational medicine.

Secondary UEDVT, which represents the majority of cases and includes our patient, occurs in patients predisposed to thrombosis due to the presence of foreign body, stasis, or hypercoagulable state. Modern physicians are increasingly using vascular access devices for parenteral antibiotics, as in our patient, as well as for chemotherapy, dialysis, parenteral nutrition, and bone marrow transplantation [8,5]. These indwelling catheters dramatically increase the risk of UEDVT. Catheter-associated venous thrombosis may occur from inappropriate positioning of the catheter tip, mechanical obstruction from precipitation of the fluid infused, or thrombosis of the catheter itself [13,14]. Factors that increase the likelihood of UEDVT include increased luminal diameter, increased number of ports, incorrect positioning, bacteremia, and prior line infection [13,14].

Surgery and casting of the upper extremity may result in stasis and significantly increase UEDVT risk, with one study finding surgery to be increasing risk 13-fold and a plaster cast increasing risk 7-fold [15]. Pacemakers and defibrillators are another significant cause of thrombosis. UEDVT occurs in 10% of placements, with increased risk in patients who have multiple leads and most events occurring within the first 2 months after device insertion [16-18]. Patients with cancer have dramatically increased risk of UEDVT. Though much of the increased risk is attributed to the frequent use of central venous catheters in this population [16], malignancy is also an independent risk factor, likely due to alteration in coagulation factors, low-grade disseminated intravascular coagulation from tumor cells, and stasis from compression by tumors [19]. Active malignancy is associated with 18-fold higher risk of UEDVT, and prior malignancy with a 7.7-fold higher risk [16]. The prevalence of hypercoagulable or prothrombotic conditions, such as Factor V Leiden or prothrombin 20210A, in patients with UEDVT ranges from 8% to 60% [14,16].

Diagnosing UEDVT rapidly and accurately is essential to prevent thrombosis-related morbidity and mortality. UEDVT may, in 25% of patients, cause post-thrombotic syndrome [20-22], which is chronic venous occlusion that may cause severe pain, swelling, intractable edema, and ulcer formation. UEDVT may also infrequently cause phlegmasia cerulean dolens, which is characterized by arterial and venous compromise and gross edema. This emergent condition requires aggressive treatment including thrombolysis or thrombectomy and carries significant risk of gangrene and limb loss [8,23]. Mortality after UEDVT is high at 15% to 50% [20,22,24]. Though this is due in part to the fact that it often occurs in patients with significant comorbidities, including malignancy, multi-organ failure, and severe infection [16,20,22,24], it also reflects the potential severity of the condition itself.

Rapid evaluation and diagnosis of venous thrombosis in patients who present to the emergency department require a modality that is easily obtained and accurate, especially considering the initiation of anticoagulation therapy or thrombolytics. Clinical signs and symptoms are often nonspecific or unreliable. Venogram, once considered the gold standard, has fallen out of favor due to its time-consuming property and its use of intravenous contrast. Compression ultrasonography has replaced this modality to be the choice of study in assessing venous thrombosis.

**Figure 6:**
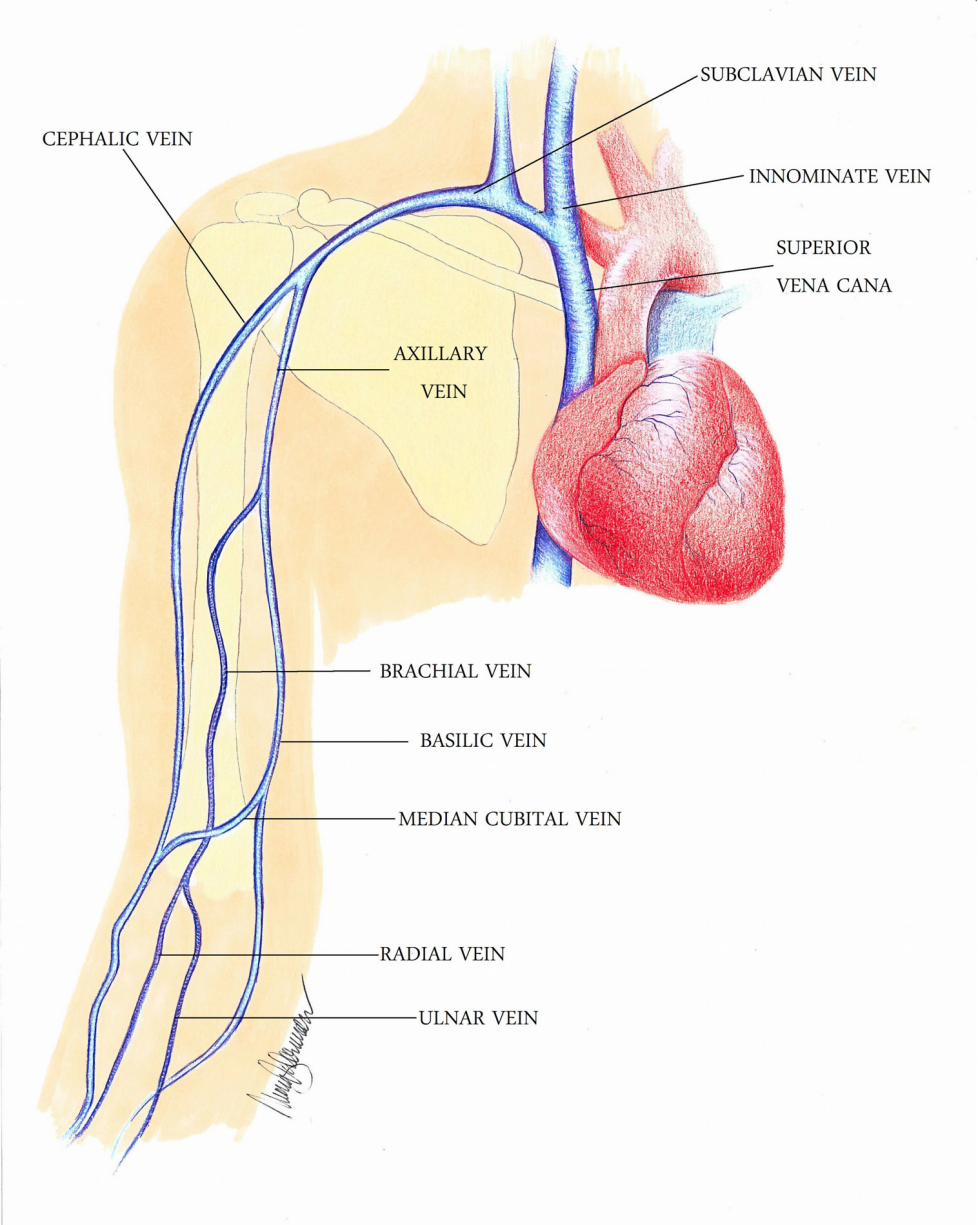
**Upper extremity veins with labels**.

Diagnosis of UEDVT using compression ultrasound is thought to be more challenging than in the lower extremity because of anatomic challenges [5]. The deep venous system of the upper extremity is comprised of paired veins below the elbow (ulnar, radial, and interosseous), which form the paired brachial veins, followed by the axillary vein, subclavian vein, and internal jugular vein, and ultimately joined to form the brachiocephalic (innominate) veins (Figure 6).

In particular, it may be difficult to visualize the proximal portion of the axillary vein due to overlying bones [5]. Despite this challenge, a recent systematic review found that compression ultrasonography has a sensitivity of 97% and a specificity of 96% [25]. To increase the accuracy, adjunct use of color Doppler and spectral waveforms may be used, especially when assessing the brachiocephalic veins [26]. Loss of pulsatility distally suggests obstructions to more centrally located veins [27].

Initial treatment of secondary UEDVT, as with our patient, includes removing the central venous catheter, and if this is not possible, the patient should receive anticoagulation treatment until the catheter can be removed and for an additional period of time [13,14]. The current standard of care for both primary and secondary UEDVT is to immediately anticoagulate with heparin and to bridge to warfarin therapy for 3 to 6 months with a target International Normalized Ratio of 2.0 to 3.0 to help maintain the patency of collateral vessels and reduce thrombus propagation [3,14].

Rapid assessment in the ED of patients with a potential UEDVT is important. Bedside ultrasonography should be followed by comprehensive radiology ultrasonography if there is diagnostic uncertainty or lack of credentialing, skill, or expertise in the performance or interpretation of the study; however, the time required for this study to be performed by a technician and officially read by a radiologist is significant in many emergency departments, particularly during nights and weekends. Swift disposition of these patients into the hospital and commencement of treatment are important for optimal patient care. In addition, once a diagnosis of UEDVT has been made in the ED, evaluation of these patients may include consideration of pulmonary embolism and other potential emergent sequela.

## Conclusion

Bedside upper extremity vascular ultrasonography can assist in the rapid diagnosis of upper extremity deep venous thrombosis in the emergency department. Rapid diagnosis of upper extremity deep venous thrombosis can expedite anticoagulation treatment.

## Consent

Written informed consent was obtained from the patient for the publication of this case report and any accompanying images. A copy of the written consent is available for review by the Editor-in-Chief of this journal.

## Competing interests

The authors declare that they have no competing interests.

## Authors' contributions

TR, BC, MK, MS, and DCR drafted and edited the manuscript. MS contributed the original artwork for the manuscript. All authors read and approved the final manuscript.

## Authors' information

DCR is the Director of Emergency Ultrasonography and Ultrasound Research. BC is the Associate Director of Emergency Ultrasonography. MK and MS are emergency department nurses at the Emergency Medicine Department, Columbia University Medical Center, New York, NY, USA. TR is an emergency medicine resident in the New York Presbyterian, Columbia/Cornell training program, New York, NY, USA.

## Supplementary Material

Additional file 1 **Emergency Department ultrasonography short-axis evaluation of the PICC Line in the brachial vein**. Video of short-axis ultrasound evaluation of a PICC line in the brachial vein. Click here for file

Additional file 2 **Emergency Department ultrasonography long-axis evaluation of the PICC Line in the brachial vein**. Video of long-axis ultrasound evaluation of a PICC line in the brachial vein. Click here for file

Additional file 3 **Emergency Department color Doppler ultrasonography short-axis evaluation of the axillary vein thrombosis**. Video of short-axis evaluation of axillary vein thrombosis. Click here for file

Additional file 4 **Emergency Department color Doppler ultrasonography long-axis evaluation of the axillary and subclavian vein thrombosis**. Video of long-axis evaluation of axillary and subclavian vein thrombosis. Click here for file

## References

[B1] Tilney ML Griffiths HJ Edwards EA Natural history of major venous thrombosis of the upper extremity Arch Surg 1970 101 792 796 10.1001/archsurg.1970.01340300148026 5489306

[B2] Ameli FM Minas T Weiss M Provan JL Consequences of "conservative" conventional management of axillary vein thrombosis Can J Surg 1987 30 167 169 3580973

[B3] Joffe HV Kucher N Tapson VF Goldhaber SZ Upper-extremity deep vein thrombosis: a prospective registry of 592 patients Circulation 2004 110 1605 1611 10.1161/01.CIR.0000142289.94369.D7 15353493

[B4] Mustafa S Stein PD Patel KC Otten TR Holmes R Silbergleit A Upper extremity deep venous thrombosis Chest 2003 123 1953 1956 10.1378/chest.123.6.1953 12796173

[B5] Blaivas M Ultrasound in the detection of venous thromboembolism Crit Care Med 2007 35 S224 S234 10.1097/01.CCM.0000260672.13913.FD 17446783

[B6] Hingorani A Ascher E Marks N Schutzer RW Mutyala M Yorkovich W Jacob T Morbidity and mortality associated with brachial vein thrombosis Ann Vasc Surg 2006 20 297 300 10.1007/s10016-006-9040-0 16779509

[B7] Kommareddy A Zaroukian MH Hassouna HI Upper extremity deep venous thrombosis Semin Thromb Hemost 2002 28 89 99 10.1055/s-2002-20567 11885029

[B8] Yanturali S Aksay E Holliman CJ Clinical pearls: left arm swelling Acad Emerg Med 2004 11 281 284 10.1111/j.1553-2712.2004.tb02210.x 15001409

[B9] Hart SD Diagnosis and management of Paget-Schroetter's syndrome Emerg Nurse 2010 18 22 25 21066922 10.7748/en2010.10.18.6.22.c8029

[B10] Rutherford RB Hurlbert SN Primary subclavian-axillary vein thrombosis: consensus and commentary Cardiovasc Surg 1996 4 420 423 10.1016/0967-2109(96)00007-5 8866074

[B11] Vijaysadan V Zimmerman AM Pajaro RE Paget-Schroetter syndrome in the young and active J Am Board Fam Pract 2005 18 314 319 10.3122/jabfm.18.4.314 15994479

[B12] Hurley WL Comins SA Green RM Canizzaro J Atraumatic subclavian vein thrombosis in a collegiate baseball player: a case report J Athl Train 2006 41 198 200 16791307 PMC1472647

[B13] Baskin JL Pui CH Reiss U Williams JA Metzger ML Ribeiro RC Howard SC Management of occlusion and thrombosis associated with long-term indwelling central venous catheters Lancet 2009 374 159 169 10.1016/S0140-6736(09)60220-8 19595350 PMC2814365

[B14] Margey R Schainfeld RM Upper extremity deep vein thrombosis: the oft-forgotten cousin of venous thromboembolic disease Curr Treat Options Cardiovasc Med 2011 13 146 158 10.1007/s11936-011-0113-1 21271312

[B15] Blom JW Doggen CJ Osanto S Rosendaal FR Old and new risk factors for upper extremity deep venous thrombosis J Thromb Haemost 2005 3 2471 2478 10.1111/j.1538-7836.2005.01625.x 16241945

[B16] Flinterman LE Van Der Meer FJ Rosendaal FR Doggen CJ Current perspective of venous thrombosis in the upper extremity J Thromb Haemost 2008 6 1262 1266 10.1111/j.1538-7836.2008.03017.x 18485082

[B17] Korkeila P Nyman K Ylitalo A Koistinen J Karjalainen P Lund J Airaksinen KE Venous obstruction after pacemaker implantation Pacing Clin Electrophysiol 2007 30 199 206 10.1111/j.1540-8159.2007.00650.x 17338716

[B18] Rozmus G Daubert JP Huang DT Rosero S Hall B Francis C Venous thrombosis and stenosis after implantation of pacemakers and defibrillators J Interv Card Electrophysiol 2005 13 9 19 10.1007/s10840-005-1140-1 15976973

[B19] Chin EE Zimmerman PT Grant EG Sonographic evaluation of upper extremity deep venous thrombosis J Ultrasound Med 2005 24 829 38 15914687 10.7863/jum.2005.24.6.829

[B20] Baarslag HJ van Beek EJ Koopman MM Reekers JA Prospective study of color duplex ultrasonography compared with contrast venography in patients suspected of having deep venous thrombosis of the upper extremities Ann Intern Med 2002 136 865 872 12069560 10.7326/0003-4819-136-12-200206180-00007

[B21] Constans J Salmi LR Sevestre-Pietri MA Perusat S Nguon M Degeilh M Labarere J Gattolliat O Boulon C Laroche JP Le Roux P Pichot O Quéré I Conri C Bosson JL A clinical prediction score for upper extremity deep venous thrombosis Thromb Haemost 2008 99 202 207 18217155 10.1160/TH07-08-0485

[B22] Prandoni P Polistena P Bernardi E Cogo A Casara D Verlato F Angelini F Simioni P Signorini GP Benedetti L Girolami A Upper-extremity deep vein thrombosis Risk factors, diagnosis, and complications. Arch Intern Med 1997 157 57 62 8996041

[B23] Sullivan VV Wolk SW Lampman RM Prager RL Hankin FM Whitehouse WM Jr Upper extremity venous gangrene following coronary artery bypass A case report and review of the literature. J Cardiovasc Surg (Torino) 2001 42 551 554 11455295

[B24] Bernardi E Pesavento R Prandoni P Upper extremity deep venous thrombosis Semin Thromb Hemost 2006 32 729 736 10.1055/s-2006-951458 17024601

[B25] Di Nisio M Van Sluis GL Bossuyt PM Buller HR Porreca E Rutjes AW Accuracy of diagnostic tests for clinically suspected upper extremity deep vein thrombosis: a systematic review J Thromb Haemost 2010 8 684 692 10.1111/j.1538-7836.2010.03771.x 20141579

[B26] Evans DS Cockett FB Diagnosis of deep-vein thrombosis with an ultrasonic Doppler technique Br Med J 1969 2 802 804 10.1136/bmj.2.5660.802 5784617 PMC1983728

[B27] Sigel B Popky GL Mapp EM Feigl P Felix WR Jr Ipsen J Evaluation of Doppler ultrasound examination Its use in diagnosis of lower extremity venous disease. Arch Surg 1970 100 535 540 10.1001/archsurg.1970.01340230001001 5438566

